# Determination of enzalutamide long-term safety and efficacy for castration-resistant prostate cancer patients after combined anti-androgen blockade followed by alternative anti-androgen therapy: a multicenter prospective DELC study

**DOI:** 10.1093/jjco/hyae004

**Published:** 2024-02-01

**Authors:** Akira Nagahara, Motohide Uemura, Mototaka Sato, Wataru Nakata, Masao Tsujihata, Tetsuya Takao, Soichi Matsumura, Kensaku Nishimura, Shingo Takada, Toshichika Iwanishi, Yasuyuki Kobayashi, Yu Ishizuya, Tsuyoshi Takada, Koichi Okada, Hitoshi Inoue, Taigo Kato, Koji Hatano, Atsunari Kawashima, Takeshi Ujike, Kazutoshi Fujita, Norio Nonomura

**Affiliations:** Department of Urology, Osaka University Graduate School of Medicine, Suita, Osaka, Japan; Department of Urology, Osaka International Cancer Institute, Osaka, Japan; Department of Urology, Osaka University Graduate School of Medicine, Suita, Osaka, Japan; Department of Urology, Fukushima Medical University, Fukushima, Japan; Department of Urology, Iwase General Hospital, Sukagawa, Fukushima, Japan; Department of Urology, Toyonaka Municipal Hospital, Toyonaka, Osaka, Japan; Department of Urology, Osaka Rosai Hospital, Sakai, Osaka, Japan; Department of Urology, Osaka Rosai Hospital, Sakai, Osaka, Japan; Department of Urology, Osaka General Medical Center, Osaka, Japan; Department of Urology, Hyogo Prefectural Nishinomiya Hospital, Nishinomiya, Hyogo, Japan; Department of Urology, National Hospital Organization Osaka National Hospital, Osaka, Japan; Department of Urology, Osaka Police Hospital, Osaka, Japan; Department of Urology, Sakai City Medical Center, Sakai, Osaka, Japan; Department of Urology, Kinki Central Hospital of Mutual Aid Association of Public School Teachers, Itami, Hyogo, Japan; Department of Urology, Higashiosaka City Medical Center, Higashiosaka, Osaka, Japan; Department of Urology, Minoh City Hospital, Minoh, Osaka, Japan; Department of Urology, Sumitomo Hospital, Osaka, Japan; Department of Urology, Ikeda City Hospital, Ikeda, Osaka, Japan; Department of Urology, Osaka University Graduate School of Medicine, Suita, Osaka, Japan; Department of Urology, Osaka University Graduate School of Medicine, Suita, Osaka, Japan; Department of Urology, Osaka University Graduate School of Medicine, Suita, Osaka, Japan; Department of Urology, Osaka University Graduate School of Medicine, Suita, Osaka, Japan; Department of Urology, Osaka University Graduate School of Medicine, Suita, Osaka, Japan; Department of Urology, Kindai University Faculty of Medicine, Osakasayama, Osaka, Japan; Department of Urology, Osaka University Graduate School of Medicine, Suita, Osaka, Japan

**Keywords:** castration-resistant prostate cancer, enzalutamide, bicalutamide, flutamide

## Abstract

**Background:**

Alternative anti-androgen therapy has been widely used as a first-line treatment for castration-resistant prostate cancer, and it may affect treatment outcome of subsequent agents targeting the androgen receptor axis. We conducted the prospective observational DELC (Determination of Enzalutamide Long-term safety and efficacy for Castration-resistant prostate cancer patients after combined anti-androgen blockade followed by alternative anti-androgen therapy) study to evaluate the efficacy of enzalutamide in patients with castration-resistant prostate cancer who underwent prior combined androgen blockade with bicalutamide and then alternative anti-androgen therapy with flutamide.

**Methods:**

The DELC study enrolled 163 Japanese patients with castration-resistant prostate cancer who underwent alternative anti-androgen therapy with flutamide following failure of initial combined androgen blockade with bicalutamide in multiple institutions between January 2016 and March 2019. Primary endpoint was overall survival. Administration of enzalutamide was started at 160 mg orally once daily in all patients.

**Results:**

The rate of decline of prostate-specific antigen by 50% or more was 72.2%, and median overall survival was 42.05 months. Multivariate analysis revealed that higher pretreatment serum levels of prostate-specific antigen (≥11.3 ng/mL; *P* = 0.004), neuron-specific enolase (*P* = 0.014) and interleukin-6 (≥2.15 pg/mL; *P* = 0.004) were independent risk factors for overall survival. Fatigue (30.0%), constipation (19.6%) and appetite loss (17.8%) were the most common clinically relevant adverse events. The enzalutamide dose was not reduced in any patient under the age of 70, but adherence was decreased in those over 70.

**Conclusions:**

In the DELC study, the safety of enzalutamide was comparable to that in previous reports. Serum levels of neuron-specific enolase and interleukin-6 were suggested as prognostic factors for castration-resistant prostate cancer with potential clinical utility.

## Teaser

We conducted the DELC study in which enzalutamide was administered after alternative anti-androgen therapy for CRPC, and median OS was 42.05 months. Higher serum levels of NSE and IL-6 were independent prognostic factors.

## Introduction

Enzalutamide is a novel agent targeting the androgen receptor (AR) axis that was initially shown in the phase III AFFIRM trial to significantly extend overall survival (OS) compared with placebo in patients with castration-resistant prostate cancer (CRPC) (1). Additionally, the phase III PREVAIL trial, which focused on pre-chemotherapy CRPC and compared enzalutamide to a placebo group, also showed the extension of OS (2,3). Based on these trial results, enzalutamide has been approved for insurance coverage in Japan and is widely used for pre-chemotherapy CRPC.

However, many CRPC cases in Japan have been treated with alternative anti-androgen therapy (AAT), which often shows long-term effects (4), but the clinical trials for CRPC to date often lacked strict criteria for the use of anti-androgens as prior therapy. Therefore, the heterogeneity of prior treatment may prevent an accurate assessment of treatment efficacy. Additionally, there are few prospective clinical studies evaluating OS in pre-chemotherapy CRPC patients, so data needed to evaluate accurate prognosis is lacking.

Therefore, we conducted the DELC (Determination of Enzalutamide Long-term safety and efficacy for Castration-resistant prostate cancer patients after combined anti-androgen blockade followed by alternative anti-androgen therapy), a prospective observational study to determine the efficacy and safety of enzalutamide in CRPC patients who underwent prior combined androgen blockade (CAB) with bicalutamide and then AAT with flutamide, and investigated the factors affecting OS.

## Patients and methods

### Study design

The study was conducted at 13 clinical sites in Japan between 26 January 2016 and 31 March 2021. The study period consisted of an enrollment period (until the end of March 2019) and 2-year follow-up period. The study was conducted in accordance with the principles of the Declaration of Helsinki and Good Clinical Practice guidelines and was approved by the institutional review board at each participating site. All patients provided written informed consent before enrollment. The study was registered at ClinicalTrials.gov (NCT02669147) and at University Hospital Medical Information (UMIN) Clinical Trials Registry (UMIN000019855).

### Patients

We recruited male patients aged 20 years or older who were receiving or had received continuous androgen deprivation therapy due to medical/surgical castration. Among them, eligible patients were those classified as 0–2 by Eastern Cooperative Oncology Group performance status, who were diagnosed as having disease progression and were treatment resistant after AAT, and who were determined eligible by the investigator for treatment with enzalutamide administration. AAT was defined as a therapy of flutamide administration after bicalutamide. The criteria for disease progression after AAT were met if one or more of the following applied: (i) prostate-specific antigen (PSA) progression during or after AAT; (ii) confirmed disease progression of soft tissue lesion as defined by RECIST v1.1; and (iii) confirmed disease progression of bone lesion defined as two or more new appearances of bone lesion on bone scintigraphy. Patients receiving or who had an administration history of enzalutamide, abiraterone, docetaxel or cabazitaxel were excluded.

### Treatments

Patients continued taking 160 mg enzalutamide orally once a day until protocol-defined progression, dose reduction, suspension or discontinuation because of adverse events (AEs), patient decline or other reason occurred during the study. Decisions on dose reduction, suspension or discontinuation were left to the discretion of each investigator. After the study regimen was discontinued, the administration of any treatment for CRPC was at the discretion of each investigator.

### Data collection

For analysis, investigators collected from the patient’s medical record observational items specified in the study protocol that included demographic and laboratory test data for entry into the electronic data capture system. The reporting schedule was defined as (i) at the screening; (ii) at enrollment; (iii) baseline (Day 0); (iv) every 3 months after enzalutamide administration (during the treatment period) and (v) at the time of discontinuation. We observed each patient until the study outcome was observed or the patient dropped out; this observation continued until March 2021. In addition, a concurrent outcome assessment was performed to survey patient survival and to confirm recovery from any serious AE 2 years after the last patient enrollment. For safety evaluation, the frequency and severity of AE were collected according to Common Terminology Criteria for AE version 4.0 criteria.

### Outcomes

The primary outcome was OS, defined as the time from the date of first dose until death from any cause. We set PSA progression-free survival (PSA-PFS), PSA response rate and compliance rate of enzalutamide as secondary outcomes. PSA-PFS was defined as the time from the date of first dose until the date of first confirmed PSA progression or death from any cause. PSA progression date was defined as the date that a ≥25% increase and an absolute increase of ≥2 ng/mL above the nadir was documented. PSA response rate was defined as the percentage of patients whose PSA level decreased by 50% or more compared with the baseline PSA level within the study period. The compliance rate of enzalutamide was calculated using the following formula:

Σ (prescribed dose per day × prescribed days)/(160 mg × total prescribed days).

### Sample size and statistical analysis

As this was a prospective observational study, we set the number of enrolled CRPC patients to 200, considering the maximum number that could be registered. For the primary analysis, the survival curve was drawn by the Kaplan–Meier method, and median survival time and its 95% confidence interval (CI) were estimated. The analysis for PSA-PFS was performed as with the primary analysis. A waterfall plot for PSA response was drawn to express the best treatment effect. Univariate analyses were performed using the Cox proportional hazards regression model to identify independent baseline factors. Subsequently, multivariate Cox regression analysis was performed using the stepwise variable selection method to narrow down independent variables and to estimate the associated hazard ratio. A *P* value < 0.05 was considered statistically significant. All statistical analyses were performed using SAS version 9.3 software (SAS Institute Inc., Cary, NC, USA) or R statistical package version Ri3863 5.3 (R Foundation, Vienna, Austria).

## Results

### Patients

**Figure 1:**
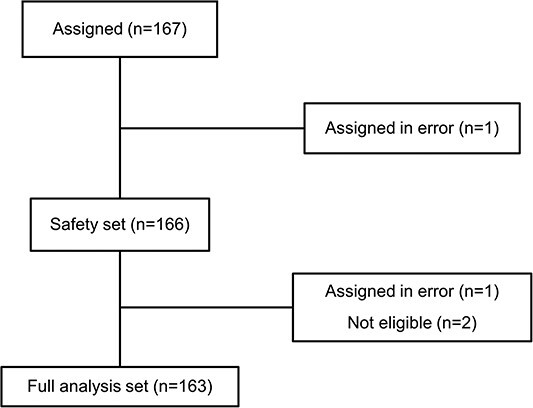
Patient disposition in the DELC study.

From 26 January 2016 to 31 March 2019, 167 patients were enrolled. Among them, one patient did not start treatment and was excluded from the safety analysis because of errors at enrollment, and three were excluded from the efficacy analysis because of errors at enrollment (*n* = 1) and ineligibility (*n* = 2) after treatment was started. Thus, 163 patients were included for efficacy analyses (Fig. 1). The median age at enrollment was 79 years old. The median serum PSA at enrollment was 11.3 ng/ml (95% CI: 0.3–928.0). The Gleason score of 125 patients (76.7%) was 8 or higher, and 120 patients (73.6%) had metastasis at enrollment. Median duration from initial diagnosis to CRPC diagnosis was 33.3 months (95% CI: 4.4–199.0). Median administration period of bicalutamide was 17.5 months (range: 0.9–175), and androgen withdrawal syndrome with bicalutamide was found in 35 patients (21.5%). Other demographic and clinical baseline characteristics are shown in Table 1.

**Table 1:** Baseline patients characteristics

Characteristics	Analysis set (*n* = 163)
Median age, year (range)	79 (60–94)
Median observation period, month (range)	25.8 (0.2–57.6)
ECOG-PS, *n* (%)	
0	131 (80.4%)
1	23 (14.1%)
2	7 (4.3%)
Gleason score at dignosis, *n* (%)	
≤7	36 (22.1%)
≥8	125 (76.7%)
Unknown	2 (1.2%)
Median serum PSA, ng/ml (range)	11.3 (0.3–928)
Metastasis at baseline, *n* (%)	
Yes	120 (73.6%)
Distribution of disease at baseline, *n* (%)	
Bone	93 (57.1%)
Pelvic lymph node	54 (33.1%)
Retroperitoneal lymph node	10 (6.1%)
Lung	14 (8.6%)
Liver	2 (1.2%)
Other	10 (6.1%)
Prior radical therapy, *n* (%)	
None	128 (78.5%)
Prostatectomy	16 (9.8%)
Radiation therapy	19 (11.7%)
Baseline hemoglobin	
Normal (≥13.7 g/dL)	27 (16.6%)
Abnormal (<13.7 g/dL)	135 (82.8%)
Unknown	1 (0.6%)
Baseline CEA	
Normal (≤5.0 ng/mL)	115 (70.6%)
Abnormal (>5.0 ng/mL)	21 (12.9%)
Unknown	27 (16.6%)
Baseline CA-19-9	
Normal (≤37.0 U/mL)	126 (77.3%)
Abnormal (>37.0 U/mL)	11 (6.7%)
Unknown	26 (16.0%)
Baseline NSE	
Normal (≤16.3 ng/mL)	120 (73.6%)
Abnormal (>16.3 ng/mL)	9 (5.5%)
Unknown	34 (20.9%)
Baseline serum teststerone, ng/mL (range)	0.14 (0.04–1.34)
Baseline serum IL-6, pg/mL (range)	2.15 (0.434–1060)
Median duration from initial diagnosis to CRPC diagnosis, month (range)	33.3 (4.4–199)
Median administration period of bicalutamide, month (range)	17.5 (0.9–175)
AWS with bicalutamide, *n* (%)	35 (21.5%)
Median administration period of flutamide, month (range)	5.8 (0.2–82.7)
AWS with flutamide, *n* (%)	4 (2.5%)

ECOG-PS, Eastern Cooperative Oncology Group performance status scale; PSA, prostate specific antigen; CEA, carcinoembryonic antigen; CA19–9, carbohydlate antigen 19–9; NSE, neuron specific enolase; IL-6, interleukin-6; CRPC, castration-resistant prostate cancer; AWS, anti-androgen withdrawal syndrome.

### Treatments

**Figure 2:**
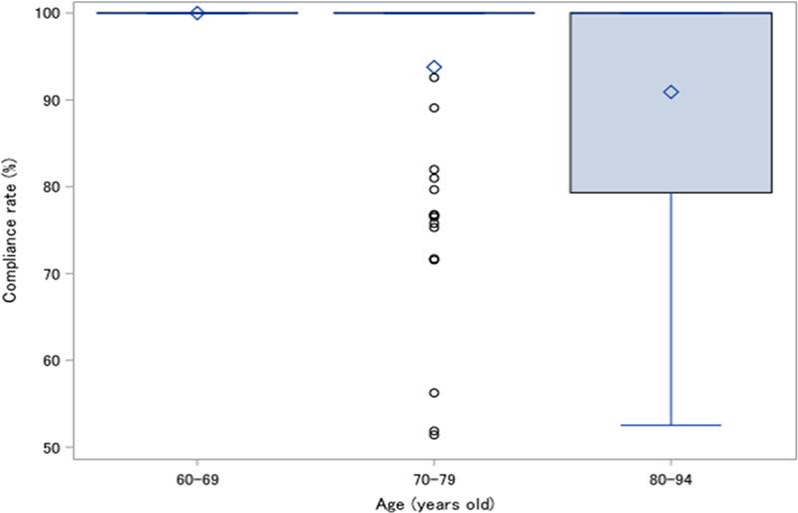
Compliance rate of enzalutamide by age group.

Among the 163 patients, 73 (44.8%) and 48 (29.4%) patients were receiving 160 mg enzalutamide at the 6- and 12-month visit, respectively (Supplementary Table S1). At the 1-month visit, 10 patients (6.8%) had reduced the dose of enzalutamide, with half of them doing so for AE (*n* = 5), and 8 patients (5.5%) had discontinued enzalutamide, with half of them also doing so for AE (*n* = 4). Moreover, at 3 months, 10 patients (7.9%) also had reduced their dose of enzalutamide, with more than half doing so for AE (*n* = 6). However, by the 12-month visit or later, no patients had reduced their dose of enzalutamide because of AE. All patients under 70 years of age completed their dose, but adherence decreased in those over 70 (Fig. 2).

### Outcomes

**Figure 3:**
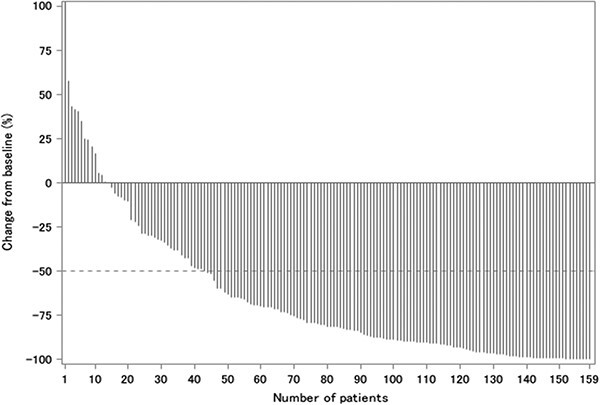
Best reduction rate of PSA by enzalutamide. PSA, prostate-specific antigen.

**Figure 4:**
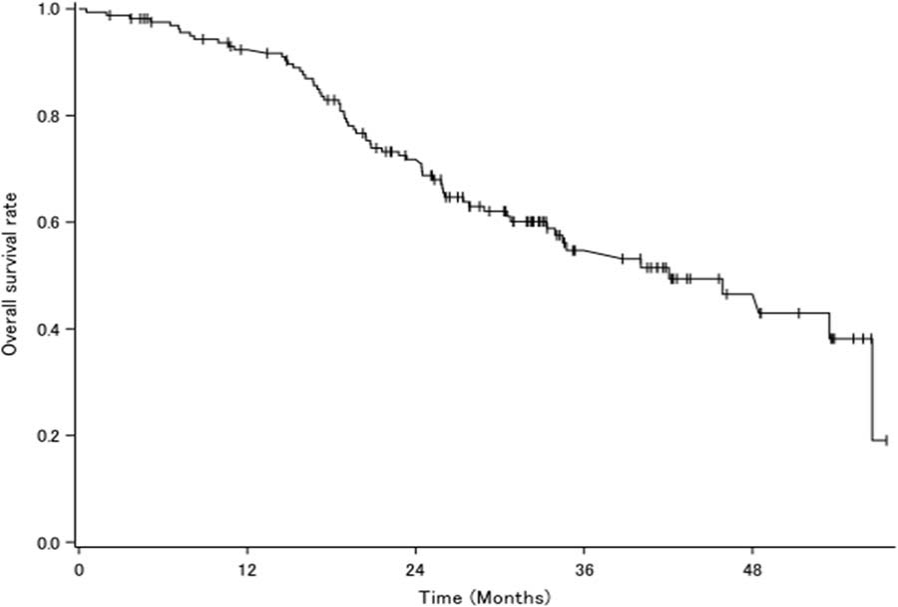
Overall survival curve for all patients.

**Figure 5:**
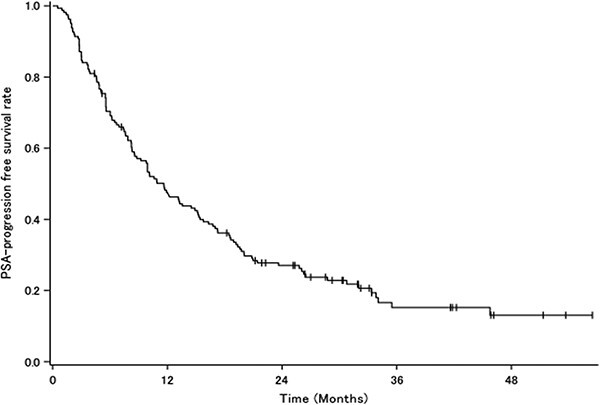
PSA-PFS curve for all patients. PSA-PFS, prostate-specific antigen progression-free survival.

The PSA response rate was 72.2%. A waterfall plot for PSA response is shown in Fig. 3. There were 72 deaths. Median OS was 42.05 months (95% CI: 33.35–56.54) (Fig. 4), and median PSA-PFS was 11.63 months (95% CI: 8.57–15.15) (Fig. 5).

### Univariate and multivariate analysis

Higher pretreatment serum levels of PSA (≥11.3 ng/mL), neuron-specific enolase (NSE) and interleukin-6 (IL-6) (≥2.15 pg/mL), and shorter duration from initial diagnosis to CRPC diagnosis (<24 months) were identified as prognostic factors for OS by univariate analysis (Table 2). In the multivariate analysis, higher pretreatment serum levels of PSA, NSE and IL-6 remained as the significant factors. The OS curve stratified by these prognostic factors is shown in Supplementary Fig. S1. Regarding PSA-PFS, higher pretreatment serum levels of PSA (≥11.3 ng/mL) and shorter duration from initial diagnosis to CRPC diagnosis (<24 months) were identified as risk factors in the univariate and multivariate analyses (Supplementary Table S2).

**Table 2:** Univariate and multivariate analysis of OS with Cox proportional hazard model

Variable	Univariate analysis		Multuvariate analysis	
	HR (95% CI)	*P* value	HR (95% CI)	*P* value
Age at treatment (≥79 years vs <79 years)	1.31 (0.81–2.12)	0.277		
ECOG-PS (≥1 vs 0)	1.75 (0.97–3.18)	0.063		
Gleason score (≥8 vs ≤7)	1.32 (0.71–2.48)	0.380		
Serum PSA at baseline (≥11.3 ng/mL vs <11.3 ng/mL)	3.03 (1.79–5.14)	<0.001	2.30 (1.30–4.10)	0.004
Metastasis (present vs absent)	1.53 (0.85–2.77)	0.157		
Baseline hemoglobin (abnormal vs normal)	0.82 (0.44–1.51)	0.522		
Baseline CEA (abnormal vs normal)	0.90 (0.42–1.89)	0.773		
Baseline CA-19-9 (abnormal vs normal)	1.14 (0.45–2.85)	0.782		
Baseline NSE (abnormal vs normal)	2.58 (1.10–6.05)	0.030	2.97 (1.24–7.11)	0.014
Baseline serum teststerone (≥0.14 ng/mL vs <0.14 ng/mL)	1.42 (0.87–2.34)	0.163		
Baseline serum IL-6 (≥2.15 pg/mL vs <2.15 pg/mL)	2.12 (1.28–3.51)	0.003	2.32 (1.32–4.09)	0.004
Duration from initial diagnosis to CRPC diagnosis (≥24 months vs <24 months)	0.40 (0.24–0.67)	<0.001	0.59 (0.34–1.03)	0.064
AWS with bicalutamide (present vs absent)	1.56 (0.88–2.79)	0.129		

OS, overall survival; HR, hazard ratio.

### Safety

The most frequently reported AEs were malaise [*n* = 49 (29.5%)], decreased appetite [*n* = 29 (17.5%)] and constipation [*n* = 19 (11.4%)]. The most frequently reported AE ≥ Grade 3 was hypertension [*n* = 7 (4.2%)]. The incidence of several AE (malaise, decreased appetite, constipation and hypertension) was higher in those aged 70–79 and ≥80 years than in those aged 60–69 years (Table 3).

**Table 3:** Overview of adverse event by age groups

	60–69 years (*n* = 24)		70–79 years (*n* = 64)		80–94 years (*n* = 78)	
	Any Grades, *n* (%)	Grade3–4, *n* (%)	Any Grades, *n* (%)	Grade3–4, *n* (%)	Any Grades, *n* (%)	Grade3–4, *n* (%)
Adverse events	8 (33.3)	0 (0)	25 (39.1)	4 (6.3)	41 (52.6)	12 (15.4)
Cardiac disorders						
Cardiac failure chronic	0 (0)	0 (0)	0 (0)	0 (0)	1 (1.3)	1 (1.3)
Gastrointestinal disorders						
Constipation	1 (4.2)	0 (0)	10 (15.6)	0 (0)	8 (10.3)	0 (0)
Nausea	0 (0)	0 (0)	4 (6.3)	0 (0)	9 (11.5)	0 (0)
Diarrhea	0 (0)	0 (0)	6 (9.4)	1 (1.6)	2 (2.6)	0 (0)
Vomiting	0 (0)	0 (0)	1 (1.6)	0 (0)	3 (3.8)	0 (0)
Ileus	0 (0)	0 (0)	0 (0)	0 (0)	1 (1.3)	1 (1.3)
General disorders and administration site conditions						
Malaise	4 (16.7)	0 (0)	20 (31.3)	1 (1.6)	25 (32.1)	0 (0)
Face oedema	0 (0)	0 (0)	1 (1.6)	0 (0)	0 (0)	0 (0)
Fatigue	0 (0)	0 (0)	0 (0)	0 (0)	1 (1.3)	0 (0)
Hepatobiliary disorders						
Hepatic function abnormal	1 (4.2)	0 (0)	2 (3.1)	0 (0)	4 (5.1)	2 (2.6)
Infections and infestations						
Kidney infection	0 (0)	0 (0)	1 (1.6)	1 (1.6)	0 (0)	0 (0)
Pyelonephritis	0 (0)	0 (0)	0 (0)	0 (0)	1 (1.3)	0 (0)
Investigations						
Platelet count decreased	2 (8.3)	0 (0)	0 (0)	0 (0)	1 (1.3)	0 (0)
Weight decreased	0 (0)	0 (0)	0 (0)	0 (0)	2 (2.6)	2 (2.6)
Metabolism and nutrition disorders						
Decreased appetite	3 (12.5)	0 (0)	10 (15.6)	1 (1.6)	16 (20.5)	1 (1.3)
Musculoskeletal and connective tissue disorders						
Muscle spasms	0 (0)	0 (0)	0 (0)	0 (0)	1 (1.3)	0 (0)
Pathological fracture	0 (0)	0 (0)	1 (1.6)	0 (0)	0 (0)	0 (0)
Nervous system disorders						
Convulsive seizure	1 (4.2)	0 (0)	3 (4.7)	0 (0)	3 (3.8)	0 (0)
Renal and urinary disorders						
Chronic renal failure	0 (0)	0 (0)	0 (0)	0 (0)	1 (1.3)	1 (1.3)
Respiratory, thoracic and mediastinal disorders						
Pneumonia aspiration	0 (0)	0 (0)	0 (0)	0 (0)	1 (1.3)	0 (0)
Skin and subcutaneous tissue disorders						
Erythema	0 (0)	0 (0)	1 (1.6)	0 (0)	0 (0)	0 (0)
Vascular disorders						
Hypertension	0 (0)	0 (0)	2 (3.1)	2 (3.1)	6 (7.7)	5 (6.4)

## Discussion

The DELC study is a single-arm prospective investigation that aimed to assess the efficacy and safety of enzalutamide in patients with CRPC. A distinct characteristic of this study is its uniform treatment history for all participants of receiving CAB with bicalutamide followed by AAT with flutamide. We have not found similar reports to date. Additionally, few studies other than clinical trials have evaluated OS as the primary endpoint in research on chemotherapy-naive CRPC. Therefore, the data obtained from the DELC study are extremely valuable.

The PREVAIL trial was also conducted in chemotherapy-naive CRPC patients, but compared with the DELC study, there were more metastatic patients (99 vs 73.6%) (2). In the enzalutamide group in the PREVAIL trial, only 21.4% of patients had received two or more anti-androgen agents prior to enzalutamide, which is less than that in the DELC study. The DELC study showed a longer median OS of 42.1 months compared with 36 months in the enzalutamide group of the PREVAIL trial. The proportion of patients with a PSA decline of ≥50% was similar between the DELC study (73.6%) and PREVAIL trial (78%), and PSA-PFS was also similar between the two studies (DELC: 11.63 months, PREVAIL: 11.2 months). However, there were differences in factors such as prior anti-androgen therapy and presence or absence of metastases between the two studies. In the subgroup analysis of metastatic cases, the OS of the DELC study was 36.01 months (Supplementary Fig. S2), which is almost the same as that in the PREVAIL trial. However, when compared with the Japanese subgroup analysis of the PREVAIL trial (5,6), OS was shorter in the DELC study, despite a similar pretreatment history, with a prior anti-androgen therapy use rate of 100%. From these results, it is possible that AAT prior to enzalutamide administration delayed the time to enzalutamide administration and may have shortened the OS after enzalutamide administration during the entire period of prostate cancer treatment.

The AFTERCAB prospective randomized controlled trial evaluated the efficacy of enzalutamide and abiraterone in chemotherapy-naive CRPC patients in Japan (7). All patients in this trial received CAB but not AAT. The rate of patients with metastases was 76.5% in the enzalutamide group, which was comparable to that of the DELC study. The primary endpoint of this study was PSA-PFS, and OS was not evaluated. The PSA-PFS of the enzalutamide group in AFTERCAB was 21.4 months, longer than that of the DELC study (11.63 months), possibly because one more lines of treatment were performed with AAT in the DELC study.

Two previous large-scale database studies reported that the adjusted OS of enzalutamide for chemotherapy-naïve CRPC to be 29.6 months (8) and 34.2 months (9). As the median OS of metastatic cases in the DELC study was 36.01 months (Supplementary Fig. S2), the results of this study are considered similar to these real-world data.

In a retrospective report of a small number of Japanese patients with CRPC after CAB treatment, Iguchi et al. (10) reported that there was no significant difference in OS between the enzalutamide administration group and the flutamide followed by enzalutamide administration group. This result supports the idea that administering flutamide as prior therapy does not affect the final OS of CRPC.

IL-6, an inflammatory cytokine, is well known as a substance that activates AR independently of ligands. IL-6 binds to the IL-6 receptor on the cell membrane and activates AR through the JAK-STAT, ERK1/2-MAPK and PI3-K pathways. Additionally, IL-6 itself is reported to promote cell proliferation and epithelial-mesenchymal transition independently of AR and is associated with the progression of CRPC (11). It was previously reported that IL-6 in the serum of prostate cancer patients is elevated both in metastatic prostate cancer patients (12,13) and in CRPC patients (14,15). George et al. (16) also reported that a high serum IL-6 level is a prognostic factor for metastatic CRPC patients. The DELC study suggested that high baseline serum IL-6 levels at the time of enzalutamide administration may lead to a poor prognosis and that IL-6 in the serum may cause the progression of prostate cancer independently of AR.

Neuroendocrine cancer is mainly composed of small-cell prostate cancer (SCPC) histologically. *De novo* SCPC is rare, accounting for only 0.5–2% of cases, and it is often diagnosed as SCPC after androgen deprivation therapy for prostate cancer and development of neuroendocrine differentiation during the course of CRPC (17). Neuroendocrine cancer is immunohistochemically negative for AR and positive for chromogranin A, NSE, synaptophysin, CD56 and other markers (18). NSE, chromogranin A and ProGRP have all been reported as serum biomarkers (19–22), and chromogranin A has also been reported as a biomarker for prognosis in CRPC in retrospective studies (22). The DELC study is the first prospective report to show that NSE is a poor prognostic factor in CRPC, although few NSE-positive cases were enrolled. Although serum IL-6 and NSE affect OS, they are not factors that affect PSA-PFS (Supplementary Table S2), so in the DELC study, they are prognostic factors for CRPC but not factors that predict the effect of enzalutamide.

Among the AE, fatigue and decreased appetite were frequent across all grades, and their frequency increased with age, whereas that of other AE was lower compared with the PREVAIL trial (2). However, AE of grade 3 or higher in both the DELC study and PREVAIL trial were mainly hypertension, which occurred in more than 5% of patients. A similar result was observed in the DELC study, with the population aged ≥80 years having a higher frequency of hypertension. Although the study started with a dose of 160 mg administered in all patients, the dose was reduced based on clinical judgment. Interestingly, the results showed that 100% of the dose was administered in the 60- to 69-year age group, but the rate of administration decreased with increasing age. Young patients up to the age of 70 can be actively treated with the full 100% dose, with fewer AE, whereas it is predicted that appropriate dose reduction will need to be implemented for older patients over the age of 70, whose frequency of AE also increases.

The DELC study has some limitations. Patients with early progression at the time of diagnosis of CRPC are considered to have a poor prognosis, and it is predicted that chemotherapy would be performed instead of AAT, so these patients considered to have a poor prognosis were not enrolled. Moreover, compared with previous reports, the duration of response to enzalutamide in the DELC study was predicted to be shorter, but this prediction is due only to subtraction of the period of AAT until enzalutamide treatment began. To prove this, it will be necessary to accumulate cases from the state of castration-sensitive prostate cancer and conduct a prospective comparative study. However, it is currently difficult to conduct such clinical research as strong treatment centered on agents targeting the AR axis is being performed for metastatic castration-sensitive prostate cancer, so there may be no other way to speculate from the results of the DELC study. Additionally, CAB is no longer recommended for prostate cancer treatment, so it is possible that this study is not consistent with current treatment. However, vintage therapy such as CAB has a history of being implemented in Japan, and CRPC patients after CAB treatment will not disappear in the future in Japan. Furthermore, CAB is a good treatment for patients who prefer inexpensive treatment. Therefore, we believe that the results of the DELC study are significant.

We investigated the efficacy and safety of enzalutamide in a homogeneous group of patients who had undergone AAT for CRPC. Safety was comparable to previous reports, and the enzalutamide response period appeared shorter due to prior AAT. NSE and IL-6 levels in serum were suggested as prognostic factors with potential clinical utility.

## Supplementary Material

Supplementary_Fig_S1_OS_stratified_by_prognostic_factors_hyae004

Supplementary_Fig_S2_OS_stratified_by_presense_of_metastasis_hyae004

Supplementary_Table_S1_hyae004

Supplementary_Table_S2_hyae004
