# Supplementary figures and images for: Determination of enzalutamide long-term safety and efficacy for castration-resistant prostate cancer patients after combined anti-androgen blockade followed by alternative anti-androgen therapy: a multicenter prospective DELC study
Source: Jpn J Clin Oncol. 2024 Feb 1;54(5):584–91. doi: 10.1093/jjco/hyae004 (PMC11075733; doi:10.1093/jjco/hyae004)

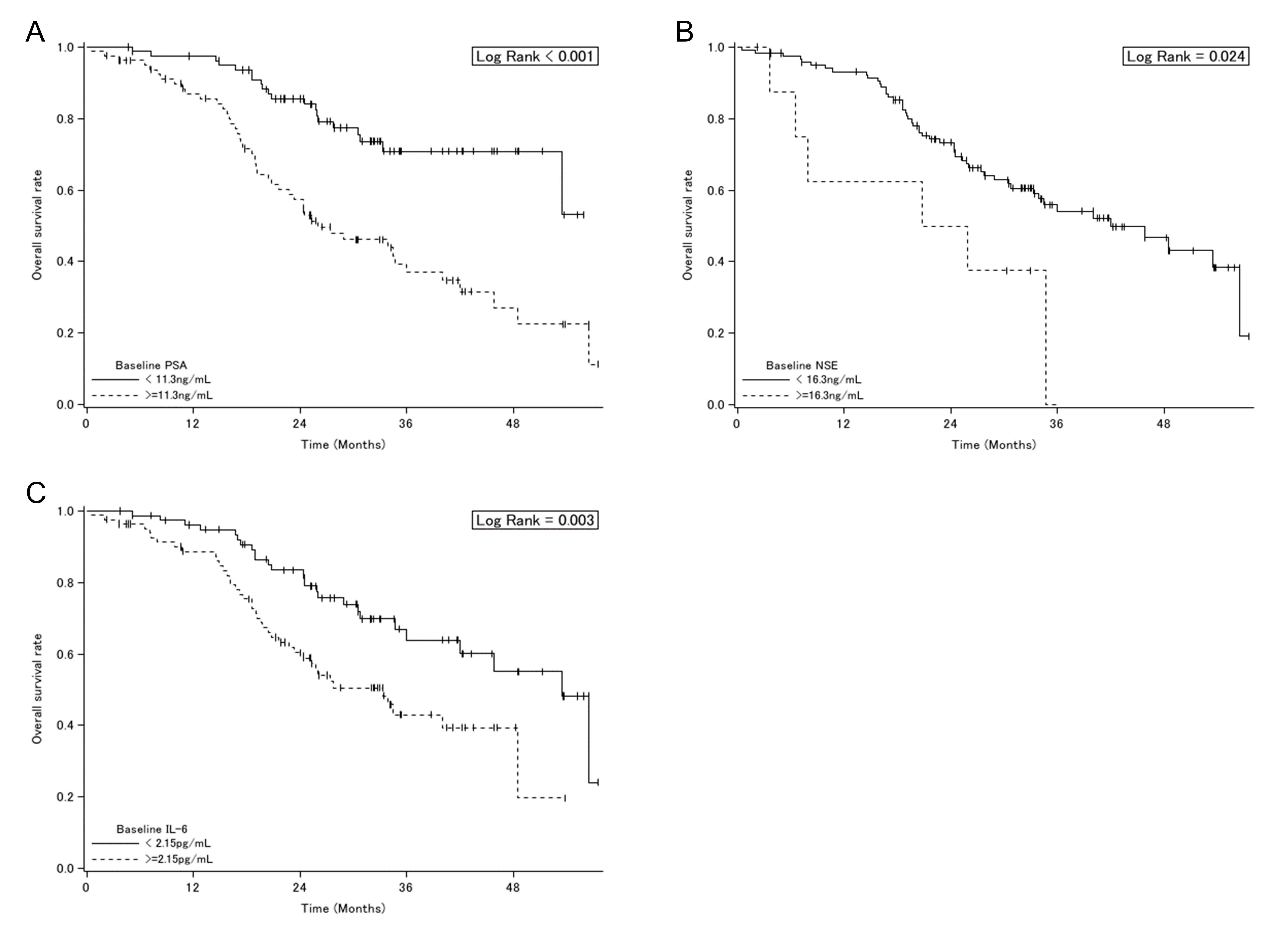

Supplement: Supplementary_Fig_S1_OS_stratified_by_prognostic_factors_hyae004 [file supplementary_fig_s1_os_stratified_by_prognostic_factors_hyae004.jpeg]

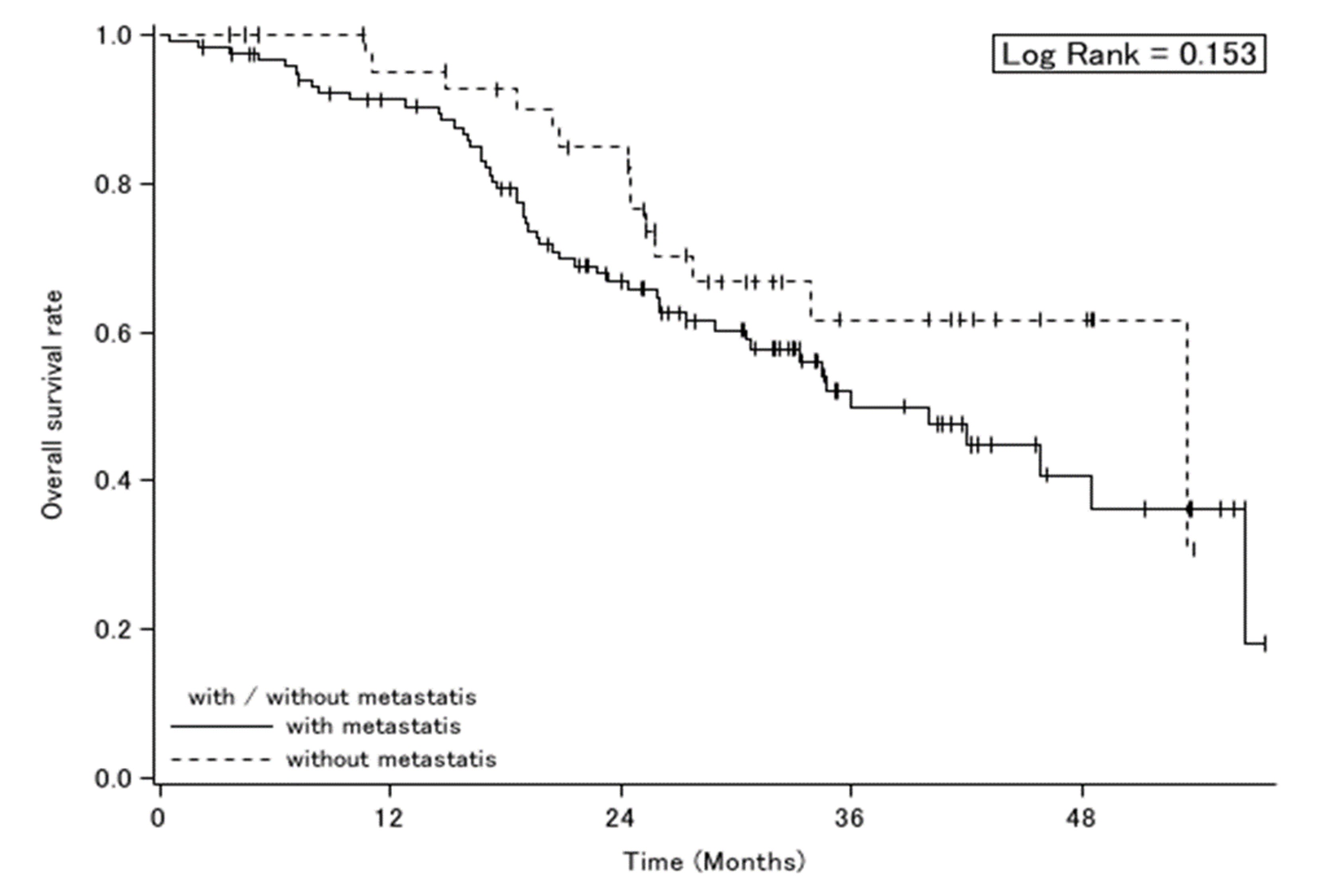

Supplement: Supplementary_Fig_S2_OS_stratified_by_presense_of_metastasis_hyae004 [file supplementary_fig_s2_os_stratified_by_presense_of_metastasis_hyae004.jpeg]
